# Comprehensive Assessment of the Health and Nutritional Status of Active-Duty United States Army Soldiers: The Military Health and Nutrition Examination Study Protocol

**DOI:** 10.1016/j.cdnut.2026.109420

**Published:** 2026-06-26

**Authors:** Claire E Berryman, Asma S Bukhari, Jennifer C Rood, David A Hughes, Adam C Lowe, Alexandra M Niclou, Meaghan E Beckner, Camila Weschenfelder, Terrence M Riley, David E Barney, Cory Dugan, Saamia B Bukhari, Faraaz H Bukhari, Michael P Smith, Brandon S Turner, Connor M Sanford, J Philip Karl, James P McClung, Tracey J Smith, Bridget A Owens, Robbie A Beyl, Steven B Heymsfield, Frank L Greenway, Stephen R Hennigar, Harris R Lieberman

**Affiliations:** 1 Pennington Biomedical Research Center, Louisiana State University, Baton Rouge, LA, United States; 2 Military Nutrition Division, U.S. Army Research Institute of Environmental Medicine, Natick, MA, United States

**Keywords:** dietary intake, nutritional surveillance, study design, NHANES, Service Members

## Abstract

**Background:**

The Military Health and Nutrition Examination Study (MHANES) is modeled after the National Health and Nutrition Examination Survey (NHANES). NHANES does not enroll active-duty Service Members, who are exposed to unique and adverse occupational challenges compared with civilians. Therefore, NHANES data are not generalizable to Service Members.

**Objectives:**

The objective of MHANES is to fill this gap by assessing dietary intake, cardiometabolic health, body composition, biomarkers of nutritional status and health, mental well-being, injury prevalence, genetics, gut microbiome composition, and physical performance in a representative sample of Army Soldiers.

**Methods:**

MHANES recruited Soldiers (n = 648) from Army installations across the United States. Participants attended an in-person visit to complete a 24-h dietary recall and questionnaires regarding demographics, physical and mental health, physical activity and performance, and dietary supplement and medication use. Height, weight, blood pressure, resting heart rate variability, physical activity, sleep, resting metabolic rate, hemoglobin mass, and blood volume were measured. Body composition was assessed using bioelectrical impedance, 3-dimensional body imaging, dual-energy X-ray absorptiometry, and circumference measurements. Blood and urine samples were collected to measure biomarkers of nutritional status, health, and genetics. Stool samples were collected to assess microbial diversity and composition. A second 24-h dietary recall was administered 3 to 10 d after the in-person visit.

**Conclusions:**

Data collected will be used to determine disease risk and prevalence and to investigate relationships between dietary intake, nutritional status, and markers of health and disease in Army Soldiers. Findings will guide evidence-based screening, education, intervention strategies, and policy decisions to improve Army health. Although the current study is focused exclusively on United States Army Soldiers, a long-term goal is to expand this research to other service branches and include additional sampling cycles similar to NHANES.

This trial was registered at clinicaltrials.gov as NCT06380322.

## Introduction

In 2009, the United States Department of Defense (DoD) established the Total Force Fitness framework across the Services to promote health and performance of active-duty Service Members (SMs) [1]. This framework consists of 8 domains, including physical fitness, social fitness, psychological fitness, environmental fitness, nutritional fitness, financial fitness, spiritual fitness, and medical and dental preventive fitness [1]. Using this framework, the Army implemented the Holistic Health and Fitness (H2F) system in 2020 to optimize physical and nonphysical performance and promote overall well-being in Soldiers [2]. However, nutritional status, cardiometabolic health, and behavioral determinants, such as dietary intake, physical activity, and sleep, are not well characterized in the entire Army population.

Past studies evaluating relationships between behavioral factors, nutritional status, and health and disease have largely been constrained to smaller, specialized populations (e.g., basic trainees, special forces), limiting generalizability to the broader Army. Therefore, there is a need for standardized assessment of dietary intake, nutritional status, physical activity, sleep, disease prevalence, and overall health and performance in a representative sample of Soldiers to characterize behavioral factors, assess disease prevalence, and examine associative relationships between behavioral factors and health and disease. These findings can then be used to inform H2F efforts and guide the design of interventions that investigate dietary modifications, new ration formulations, weight management strategies, and pharmacotherapies to promote health.

The current study, the Military Health and Nutrition Examination Study (MHANES), is modeled after the NHANES conducted by the Centers for Disease Control and Prevention, in collaboration with the NIH, the National Center for Health Statistics, and the USDA [3]. NHANES assesses the nutritional, biochemical, and medical status of a representative sample of the United States population. NHANES is essential for the nation’s health information system because it collects data directly from participants through clinical examinations and medical and laboratory testing, in conjunction with self-reported data. However, NHANES does not enroll active-duty SMs. Furthermore, NHANES data are not generalizable to SMs because they are often exposed to more adverse occupational challenges than the civilian population, such as long work hours, extensive physical demands, deployment, separation from family members, and austere environments. Therefore, comprehensive information on the nutritional, biochemical, and medical status of a representative sample of active-duty SMs is not currently available. MHANES is designed to address this research gap by assessing nutritional, biochemical, and medical status and lifestyle behaviors in a representative sample of active-duty Army Soldiers.

## Trial aim

The aim of MHANES is to provide comprehensive information on nutritional, biochemical, and medical status and lifestyle behaviors of active-duty Army Soldiers by assessing dietary intake, cardiometabolic health, body composition, biomarkers of nutritional status and health, mental well-being, sleep, physical activity, injury prevalence, genetics, gut microbiome composition, and physical performance in a large representative sample of Army Soldiers.

## Methods

### Institutional review board approval and trial registration

The study protocol was approved by the Institutional Review Board at Pennington Biomedical Research Center (PBRC) and the United States Army Medical Research and Development Command (USAMRDC) Office of Human Research Oversight. The clinicaltrials.gov identifier is NCT06380322.

### Study population and trial design

In this cross-sectional study, data were collected from August 2024 to February 2026 in active-duty Army Soldiers (*n* = 648) from the following 5 geographically dispersed Army installations in the United States: Fort Polk, Louisiana; Fort Campbell, Kentucky; Joint Base Lewis-McChord, Washington; Fort Carson, Colorado; Fort Stewart, Georgia (Table 1). These Army installations represent a convenience sample because selection was based on the availability of a letter of support from the Commanding General at each installation. Marketing efforts at each installation were as broad as possible to ensure adequate sampling across demographic subgroups of sex, race/ethnicity, and grade/rank and, ultimately, to recruit a representative sample of active-duty Army Soldiers. In addition, we oversampled females and certain race/ethnicity groups to ensure that these groups are adequately powered for analyses (Table 2). For example, females were oversampled because they are a smaller proportion of the Army population and are at greater risk of several nutritional deficiencies including iron and iodine [4,5].

**TABLE 1:** Data collection location, dates, and enrollment for the Military Health and Nutrition Examination Study (MHANES) population

Location	Date	Enrollment
Fort Polk, LA	August to September 2024	126
	January to February 2026	38
Fort Campbell, KY	September to October 2024	134
Joint Base Lewis-McChord, WA	January to February 2025	137
Fort Carson, CO	March to April 2025	123
Fort Stewart, GA	July to August 2025	90

**TABLE 2:** Oversampling plan for the Military Health and Nutrition Examination Study (MHANES) population

	Percentage of the Army population^1^	Percentage of the MHANES population^2^
Sex		
Female	16	32
Male	84	68
Race/ethnicity		
American Indian or Alaskan	2	4
Native/other/unknown		
Asian or Pacific Islander	7	14
Non-Hispanic Black	21	21
Hispanic	17	17
Non-Hispanic White	53	44
Rank		
Enlisted	82	82
Officer	18	18

1 Based on demographics from 2021.

2 Target demographics for MHANES.

Post-stratification survey weights were applied to align the analytic sample with the demographic composition of the active-duty Army population based on sex, race/ethnicity, and rank. Although these weights do not explicitly account for site-based recruitment, they help mitigate potential bias to the extent that differences across installations are reflected in these characteristics. In addition, active-duty Soldiers routinely relocate between installations approximately every 3 to 4 y, resulting in broadly similar demographic distributions across sites. Steps to minimize sampling bias included geographic dispersion of installations, broad recruitment strategies at each site, and balanced enrollment targets across installations (∼100–150 participants at each site).

Interested Soldiers attended an in-person briefing to learn about the study and, if still interested in participating, signed the informed consent before any data collection. The study inclusion and exclusion criteria are shown in Table 3. After signing the informed consent, volunteers are asked to complete a screening questionnaire to obtain demographic information (i.e., sex, age, race/ethnicity, rank/grade, area of assignment, military occupational specialty, and duration at current duty station). Volunteers were then given instructions (i.e., no alcohol for 48 h, no exercise or caffeine for 12 h, no food or beverages except water for 10 h, and no tobacco or nicotine products in the morning before the visit) and scheduled for 1 in-person visit (∼4 h) where outcome measures were assessed (see below). A stool sample kit was provided to volunteers so they could collect the sample at home and bring it to the study visit. Volunteers were also provided with a bag to carry their dietary supplements and medications to the study visit. A subset of volunteers received an actigraph watch to monitor sleep and physical activity to wear until their in-person visit (∼4–7 d after the briefing visit).

**TABLE 3:** Inclusion and exclusion criteria for the Military Health and Nutrition Examination Study

Inclusion criteria	Exclusion criteria
• United States Army SMs ≥18 y • Willing to have biological samples stored for future use • Willing to have data linked to the Soldier Performance, Health, and Readiness (SPHERE) database	• Inability to understand verbal or written instructions in English • SMs relocating or getting out of the Army in the next 30 d • Pregnant • Currently in Basic Combat Training(BCT) and/or One-station Unit Training (OSUT)

Abbreviation: SM, Service Member.

### Outcome measures

Study personnel were trained to collect data in accordance with standardized protocols to ensure consistency and accuracy across the study.

#### Anthropometrics

Body weight was assessed in the morning (06:30–11:00), after an overnight fast, after first toilet use, and in light clothing using a bioelectrical impedance device (InBody 770; InBody USA). Height was measured in duplicate to the nearest 0.1 cm using a stadiometer (Seca; Creative Health Products).

#### Body circumferences

Neck, waist (iliac crest), and hip circumference were measured using a nonstretchable tape measure (Gullick II Tape; Creative Health Products) based on the NHANES anthropometry and Army Regulation AR600-9 methods [6,7]. Circumference measurements were taken 3 times and recorded to the nearest 0.1 cm. If the measurements were within 1 cm of each other, the mean was taken. If any 1 of the 3 measurements differed by >1 cm, an additional measurement was taken. Then, the 3 closest measures were averaged.

#### Body circumferences and composition using 2D/3D body imaging

Body circumferences and composition were also estimated using an optical imaging system (Mobile Fit Booth; Size Stream LLC). The participant stood on a stationary platform while 1 image was taken in each of 2 positions (facing the camera and a side view) using a tablet camera. The proprietary software derived circumference measurements from a 3-dimensional avatar of the participant.

#### Body composition using bioelectrical impedance

Fat mass, body fat percentage, fat-free mass, skeletal muscle mass, and total body water were measured using bioelectrical impedance (InBody 770; InBody USA).

#### Body composition measured using dual-energy X-ray absorptiometry

At installation(s) with an accessible dual-energy X-ray absorptiometry (DXA) machine (i.e., Fort Stewart), all participants (*n* = 90) were asked to undergo a DXA scan. Body composition (total body weight, fat-free mass, fat mass, and bone mass) was determined after an overnight fast (Lunar Prodigy; General Electric). A pregnancy test was administered to all females before the DXA scan to confirm that they were not pregnant.

#### Heart rate variability

Resting heart rate variability (HRV) was examined according to the Task Force for Pacing and Electrophysiology guidelines [8]. Heart rate variations were recorded with the Zephyr Bioharness (Zephyr Technology Corporation), a wireless chest-worn device that allows heart rate, respiratory rate, core temperature, activity levels, and posture to be recorded in real time. Volunteers were asked to lie in a supine position and remain motionless and awake for ∼20 min in a quiet area while wearing the Zephyr Bioharness. Resting HRV with spontaneous breathing was collected from minutes 05:00 to 12:00. Participants were then asked to breathe at an inhalation:exhalation rate of 5 s:5 s by following an audible metronome, and resting HRV with paced breathing was collected from minutes 13:00 to 20:00. Time- and frequency-domain and nonlinear components of HRV will be calculated and analyzed using Kubios software (available at http://kubios.uef.fi) [9].

#### Indirect calorimetry

Fasted resting metabolic rate was measured using indirect calorimetry (BodyGem; Microlife Medical Home Solutions, Inc.). Before assessment, participants rested in the supine position for ≥20 min in a quiet and dimly lit room. During the measurement, participants sat quietly with their feet flat on the floor. Participants were asked to breathe into a disposable mouthpiece connected to the indirect calorimeter until steady-state oxygen consumption was reached (5–10 min). The indirect calorimeter measures oxygen consumption, which is converted to calories using the Weir equation and a constant respiratory quotient value of 0.85 [10].

#### Resting blood pressure

After ≥5 min of seated rest, blood pressure was measured with an automated cuff (Omron HEM-907XL; Omron Healthcare, Inc.). Three measurements were taken, separated by ≥1 min, and averaged to calculate resting blood pressure.

#### Hemoglobin mass and blood volume

Hemoglobin mass (Hbmass) was determined in a subset of participants (*n* = 230) using the automated carbon monoxide (CO)-rebreathing method (Detalo Health). The amount of time to complete measurements (∼10–15 min/participant) and the availability of equipment and supplies limited the number of participants able to complete this measure. The method is based on the dilution principle, where a small volume of tracer (CO) is administered, and the subsequent concentration of carboxyhemoglobin (COHb) in the blood can be used to calculate Hbmass. Red blood cell volume, plasma volume, and total blood volume can then be calculated from Hbmass. CO was administered to participants in combination with 100% O_2_. The gas mixture was then rebreathed within a closed system for 4 min. Before and 6 min after CO-rebreathing, arterialized capillary blood was collected from a fingertip to assess changes in COHb (%) using a blood gas analyzer (OSM3; Radiometer). Consistent with previous methods [11], each blood sample was measured in triplicate. If the results differed by >0.1%, 2 additional samples were collected (for a total of 5).

#### Physical activity and sleep

Physical activity and sleep data were collected by actigraphy (Actigraph GT3X+; ActiGraph) in a self-selected subset of participants (*n* = 174). Actigraphy assessments were optional and based on device availability. The actigraph was worn on the nondominant wrist for ≥5 d. Acceleration data were recorded every 15 s and computed using the Freedson Adult thresholds to estimate metabolic equivalents and step counts and identify activity type, such as sedentary, light, moderate, vigorous, and very vigorous activity [12]. Adherence to the activity monitor was obtained through the “wear time” function in ActiLife software (ActiGraph). Data from the first and last day of wear were not included in the analysis, and 3-d averages were computed for participants with ≥3 d of >10 h of awake time data.

For sleep analyses, raw actigraphy data were saved as 60-s epoch files and imported into ActiLife using the batch sleep processing feature. The Cole–Kripke sleep algorithm [13] and the Tudor-Locke algorithm [14] were applied using default settings to detect sleep periods. Averages for total sleep time, sleep efficiency, and wake after sleep onset were calculated from 3 d of wear time (i.e., days 2–4). Participants were asked to record information related to their sleep habits on the days they wore the accelerometer.

#### Doubly labeled water method

A subset of participants (*n* = 106) was dosed with doubly labeled water to assess total daily energy expenditure (TDEE). TDEE assessment, an optional study measure, was collected in as many participants as feasible within logistical and budgetary constraints. Before dosing, volunteers provided a urine sample that was used to measure background isotope enrichment. The participant then consumed 1.5 g/kg body weight of a mixture of 1.425 g 10% H_2_^18^O per kg body weight and 0.075 g 99.8% ^2^H_2_O per kg body weight (Sigma-Aldrich). The container was rinsed with ∼50 mL bottled water for the participant to then consume. Urine was collected at 4.5 h and 7 d after dosing. Isotopic elimination rates were calculated using regression and the equation developed by Speakman et al. [15] to assess the rate of carbon dioxide (CO_2_) production. Energy expenditure was calculated by multiplying the rate of CO_2_ production by an assumed food quotient of the diet of 0.86.

#### Blood measures

Fasted venous blood samples (54 mL) were collected by a credentialed phlebotomist at the in-person visit. Blood parameters that will be measured are shown in Table 4. Genetic variation at 1.8 million markers (e.g., single-nucleotide polymorphisms) will be measured from serum blood samples using Illumina’s Infinium Global Diversity Array genotyping platform. We chose this array, along with our downstream imputation pipeline, because it is used by and can be harmonized with the All of Us dataset, uses a multiancestry array design, has content breadth, and can be imputed to a diverse genome reference. Zinc protoporphyrin was measured immediately after collection using the ProtoFluor Z Analyzer (Helena Laboratories). Complete blood cell counts were analyzed in whole blood within 48 h of blood collection. Plasma, serum, and whole blood were divided into aliquots in prespecified tubes to limit the occurrence of freeze–thaw cycles and shipped on dry ice for storage at −80°C before analysis. After initial assays are completed, the remaining blood will be archived and retained for future use.

**TABLE 4:** Blood biomarkers assessed in the Military Health and Nutrition Examination Study

Measures of anemia and iron status	Nutrient status and electrolytes	Hormone status	Biomarkers of stress	Cardiometabolic health and inflammation
Hemoglobin	Calcium	Total testosterone	Cortisol	Glucose
Hematocrit	25-OH vitamin D	Estradiol	DHEAS	Hemoglobin A1c
Red cell distribution width	Vitamin D–binding protein	Sex hormone–binding globulin	Creatine phosphokinase	Insulin
Mean cell hemoglobin concentration	Magnesium	Thyroid-stimulating hormone		Lipids (total cholesterol, non-HDL-C, LDL-C, HDL-C, and triglycerides)
Mean cell hemoglobin	Chloride	Triiodothyronine		Amylase
Mean cell volume	Sodium	Thyroxine		Liver enzymes
Ferritin	Phosphorus	PTH		hsCRP
sTfR	Potassium			α-1-Acid glycoprotein
Iron	Vitamin B-12			Complete blood cell count
TIBC	Folate			Albumin
Hepcidin	RBC fatty acids			Total protein
Erythropoietin	Retinol binding protein			Blood urea nitrogen
Erythroferrone				Creatinine
Zinc protoporphyrin				

Abbreviations: DHEAS, dehydroepiandrosterone sulfate; hsCRP, high-sensitivity C-reactive protein; PTH, parathyroid hormone; RBC, red blood cell; sTfR, soluble transferrin receptor; TIBC, total iron–binding capacity.

#### Urinary measures

Participants provided a fasted urine sample (second void of the day). Urine was divided into aliquots in prespecified tubes to limit the occurrence of freeze–thaw cycles and was shipped on dry ice for storage at −80°C before analysis. Iodine, creatinine, and a semiquantitative measure of microalbumin (i.e., the albumin-to-creatinine ratio) will be measured in the urine sample. After initial assays are completed, the remaining urine will be archived and retained for future use.

#### Gut microbiome diversity and composition

Stool samples were collected by participants into collection tubes containing DNA/RNA shield (#R1101; Zymo Research). Participants were instructed to store and transport samples at room temperature and deliver them to study staff within 7 d of collection. Upon receipt by study staff, samples were frozen and shipped at −20°C. Once samples arrived at PBRC, they were stored at −80°C until analysis. Genomic DNA will be extracted using standard bead-based extraction methods and quantified before analysis. Shotgun metagenomic sequencing of extracted DNA will be conducted for taxonomic and functional profiling of the gut microbiome.

#### Psychomotor Vigilance Test (National Aeronautics and Space Administration PVT+)

This is a 5-min test of simple visual reaction time widely used to assess alertness [16]. The test requires volunteers to sustain attention and respond to stimuli presented at random intervals on a computer screen by pressing a button. Parameters recorded include reaction time, premature responses, correct responses, and number of lapses. This test was completed while participants were in the fasted state to minimize the acute effects of dietary intake, caffeine, and nicotine on reaction time.

#### Questionnaires

Questionnaires assessed demographic and lifestyle information, eating behaviors and diet quality, current and past physical and mental health, prescription drug and dietary supplement use, and physical performance on military fitness tests (Table 5). Questionnaires were administered at the in-person visit and required ∼90 to 120 min to complete. Questionnaire data were collected via a tablet device and managed using Research Electronic Data Capture (REDCap) electronic data capture tools at PBRC [17,18].

**TABLE 5:** Questionnaires administered in the Military Health and Nutrition Examination Study

Questionnaire/instrument	Domain assessed	Items (no.)	Estimated time (min)	Key variables/outcomes
Lifestyle and Health Questionnaire	Lifestyle behaviors, general health	72	15–20	Tobacco/alcohol use, medical conditions, physical activity behaviors, income
Military Eating Behaviors Survey (MEBS)	Eating behaviors and mediators	133	30–40	Hunger, satiety, cravings, meal patterns, restraint, diet rigidity, emotional eating, food choice, stress, food access
Patient Health Questionnaire (PHQ-9)	Depression	9	2–3	Depression severity score (0–27)
Generalized Anxiety Disorder (GAD-7)	Anxiety	7	2–3	Anxiety severity score (0–21)
Life Events Checklist for DSM-5 (LEC-5)	Trauma exposure	16	3–5	Exposure to traumatic events
Primary Care PTSD Screen for DSM-5 (PC-PTSD-5)	PTSD	5	2–3	PTSD risk (score 0–5)
General Self-Efficacy Scale (GSE)	Self-efficacy	10	2–3	Self-efficacy score (10–40)
Pittsburgh Sleep Quality Index (PSQI)	Sleep quality	19	5–7	Sleep duration, efficiency, disturbances, total sleep score
Connor-Davidson Resilience Scale (CD-RISC)	Resilience	25	5–7	Resilience score (0–100)
Profile of Mood States (POMS 2-A)	Mood states	65	12–15	Tension, depression, anger, vigor, fatigue, confusion, total mood disturbance
Dimensional Obsessive-Compulsive Scale (DOCS)	OCD symptom severity	20	5–7	OCD symptom domains (contamination, symmetry, harm, taboo thoughts)
Injury Questionnaire	Musculoskeletal injury history	∼10–15 (variable)	3–10	Injury type, location, date of injury, severity, cause, and duration
Pain, Enjoyment, General Activity (PEG) Scale	Pain and function	3	1–2	Pain intensity and interference (0–10)
Reproductive Health Questionnaire (females only)	Reproductive health	∼30–40 (variable)	10–15	Menstrual status, contraceptive use, reproductive history

Abbreviations: DSM-5, Diagnostic and Statistical Manual of Mental Disorders, 5th edition; OCD, obsessive-compulsive disorder; PTSD, post-traumatic stress disorder.

#### Lifestyle and Health Questionnaire

Demographic, lifestyle, and health information, including but not limited to age, education, marital status, race/ethnicity, alcohol and tobacco use, food security, and physical activity, was assessed via questionnaire. Reproductive health information was collected from females via a separate questionnaire.

#### Military Eating Behaviors Survey

The Military Eating Behaviors Survey (MEBS) is a validated tool to examine military eating habits and mediators of eating behavior and was administered at the in-person study visit [19]. MEBS has 133 items (43 eating habits; 90 mediating behaviors), with 14 eating habit scales (hunger, satiety, food craving, meal pattern, restraint, diet rigidity, emotional eating, fast/slow eating rate, environmental triggers, situational eating, supplement use, and food choice) and 8 mediating factor scales (body composition strategy, perceived stress, food access, sleep habits, military fitness, physical activity, military body image, and nutrition knowledge).

#### Patient Health Questionnaire-9

The Patient Health Questionnaire-9 (PHQ-9) is a 9-item, self-reported depression questionnaire used by health professionals to identify potential cases of major depressive disorder or other depressive disorders. The PHQ has been shown to be a valid measure of depression severity [20]. This questionnaire identifies feelings over the past 2 wk based on a 4-point scale (ranging from “Not at all” to “Nearly every day”). The PHQ-9 questionnaire is scored from 0 to 27; higher scores represent greater depression severity. For the following question: “Over the last 2 wk, how often have you been bothered by the following problem: Thoughts that you would be better off dead or of hurting yourself in some way?”, if a participant answers “SEVERAL DAYS,” “MORE THAN HALF THE DAYS,” or “NEARLY EVERY DAY,” the individual was referred to the Command’s Behavioral Health Officer or Emergency Medical/Behavioral Health Services.

#### General Anxiety Disorder 7-item scale

Anxiety was assessed with the Generalized Anxiety Disorder 7-item scale (GAD-7), a validated measure of anxiety used to identify anxiety disorders [21]. The GAD-7 is composed of 7 items and is scored from 0 to 21; higher scores represent greater anxiety.

#### The Life Events Checklist for Diagnostic and Statistical Manual of Mental Disorders, 5th edition

The Life Events Checklist for Diagnostic and Statistical Manual of Mental Disorders, 5th edition (LEC-5) was administered to participants to screen for exposure to potentially traumatic events [22]. The LEC-5 consists of 16 events strongly associated with the development of post-traumatic stress symptoms.

#### Primary care post-traumatic stress disorder screen for Diagnostic and Statistical Manual of Mental Disorders, 5th edition

The primary care post-traumatic stress disorder (PTSD) screen for Diagnostic and Statistical Manual of Mental Disorders, 5th edition (PC-PTSD-5) is a self-report 5-item questionnaire used to identify individuals with probable PTSD. The screener begins with a question to assess whether the respondent has had any exposure to traumatic events. If a respondent denies exposure, the PC-PTSD-5 is completed with a score of 0. If a respondent indicates a trauma history—experiencing a traumatic event over the course of their life—the respondent answered 5 additional yes/no questions about how that trauma has affected them over the past month. Respondents can score 0 to 5. The PC-PTSD-5 was validated in a large sample of Veterans Affairs primary care patients [23].

#### General Self-Efficacy Scale

The General Self-Efficacy Scale (GSE) is a 10-item self-report measure of self-efficacy. The GSE is positively correlated with optimism and work satisfaction and negatively correlated with depression, stress, health complaints, burnout, and anxiety [24]. The total score ranges between 10 and 40, with a higher score indicating greater self-efficacy.

#### The Pittsburgh Sleep Quality Index

Sleep quality was assessed with the Pittsburgh Sleep Quality Index (PSQI). The PSQI is a validated scale used to assess duration of sleep, sleep disturbance, sleep latency, daytime dysfunction due to sleepiness, sleep efficiency, overall sleep quality, use of hypnotics, and total sleep quality index [25]. The PSQI is composed of 19 items and is scored from 0 to 21 (sum of 7 component scores from 0 to 3); higher scores represent greater sleep difficulties.

#### Connor-Davidson Resilience Scale

Resilience was assessed with the Connor-Davidson Resilience Scale (CD-RISC), which has been validated to assess resilience in general and clinical populations [26]. The CD-RISC is composed of 25 items and is scored from 0 to 100; higher scores represent higher levels of resilience.

#### Profile of Mood States 2nd Edition–Adult

Mood was assessed with the Profile of Mood States 2nd Edition–Adult (POMS 2-A), a widely used psychological inventory of mood states [27]. Volunteers rated 65 mood-related adjectives describing how they felt over the past week, which were used to generate 6 factor-analytically derived subscale scores (tension, depression, anger, vigor, fatigue, and confusion) as well as a total mood disorder score. Higher scores indicate greater mood disorder. This questionnaire was completed while participants were in a fasted state to minimize the acute effects of dietary intake, caffeine, and nicotine on mood.

#### Dimensional Obsessive-Compulsive Scale

Obsessive-compulsive disorder (OCD) symptom severity was assessed using the Dimensional Obsessive-Compulsive Scale, a 20-item self-report instrument that assesses the OCD symptom severity along the following 4 dimensions: contamination; responsibility for harm and mistakes; incompleteness/symmetry; and unacceptable (taboo) thoughts [28].

#### Injury Questionnaire

Volunteers were asked to report musculoskeletal injuries incurred in the last 6 mo. For each injury, they were asked the type, location, side of the body, date of injury, severity of injury, cause of injury, and duration of the injury.

#### The Pain, Enjoyment of Life, and General Activity Scale

The Pain, Enjoyment of Life, and General Activity (PEG) scale is a brief, 3-item, participant-reported pain outcome measure derived from the Brief Pain Inventory [29]. The PEG scale includes 3 questions assessing pain intensity and functional interference (i.e., interference with enjoyment of life and general activity). The PEG scale is scored by averaging the 3 items; scores range from 0 to 10.

#### Twenty-four hour dietary recall assessment

Twenty-four hour dietary recalls were collected using Automated Self-Administered 24-Hour Dietary Recall (ASA24) (https://epi.grants.cancer.gov/asa24/), providing estimates of macronutrient (protein, carbohydrate, and fat) and micronutrient (vitamins and minerals) intake. ASA24 is a validated and standardized web-based tool that assesses food, beverage, and dietary supplement consumption in the previous 24 h. ASA24 is automated and self-administered and allows individuals to record what food and dietary supplements they consumed without the assistance of an interviewer, thereby minimizing respondent bias. This study collected one ASA24 dietary recall at the in-person visit and a second 24-h dietary recall 3 to 10 d later electronically via a personal device.

#### Medication and supplement interview

Volunteers were asked about their prescription, over-the-counter medication, and dietary supplement use over the previous 30 d. Participants were asked to bring in or take a picture of each medication and dietary supplement they were currently taking to review with research staff at the in-person visit. Volunteers were asked the name, dose, unit, frequency, route, indication, and start/stop date for each medication and dietary supplement.

#### Soldier Performance, Health, and Readiness database linking

The Soldier Performance, Health, and Readiness (SPHERE) database is a secure data repository that combines United States Army population data from multiple distinct DoD agencies. It is housed and managed at the United States Army Research Institute of Environmental Medicine by a team of epidemiologists, analysts, and database managers [30]. Retrospective and prospective data pertaining to medical events, changes in demographics, performance parameters, and base assignments and deployments may be requested from the SPHERE database.

### Data quality control and management

Procedures have been implemented to ensure data quality, harmonization, and integrity across study sites and assessment modalities. All study personnel completed standardized training and certification procedures before participant enrollment, and detailed standard operating procedures were followed for all assessments and biospecimen handling procedures. Periodic retraining and monitoring of protocol adherence were conducted throughout the study. Body circumference and blood pressure measurements were collected in triplicate using standardized protocols to minimize measurement error and improve reliability.

The majority of the questionnaire data were collected and managed using REDCap electronic data capture tools hosted at PBRC. REDCap incorporates audit trails documenting data modifications and role-based access controls to enhance data accuracy and security. Dietary recall, psychomotor vigilance, and POMS data were collected via the ASA24 website, National Aeronautics and Space Administration PVT+ application, and the Multi-Health Systems Online Assessment Center, respectively. Medication and dietary supplement use data were recorded on paper forms and subsequently entered into electronic spreadsheets. Data from these platforms will be securely downloaded and integrated into a centralized data repository for harmonization, quality control, and analysis.

Certain physiological data collected during in-person assessments (e.g., body circumferences, resting metabolic rate, and blood pressure) were recorded on paper forms and subsequently entered independently by 2 study staff members into electronic spreadsheets. A third staff member verified agreement between entries and reconciled discrepancies against the original source documents to minimize transcription errors. Body composition, HRV, and Hbmass and blood volume data were directly downloaded from the respective equipment. Data will be integrated into a centralized data repository for harmonization, quality control, and analysis.

Biospecimens are processed using standardized protocols and tracked using unique participant identifiers. Whole blood, plasma, serum, urine, and stool samples were divided into aliquots in prespecified tubes to minimize freeze–thaw cycles before long-term storage. Laboratory analyses are centralized to minimize interlaboratory variability. Instrument calibration and quality control procedures are conducted according to the manufacturer’s recommendations.

Before analysis, datasets will undergo centralized harmonization and cleaning procedures, including standardized variable naming conventions, coding dictionaries, review of outliers and implausible values, and cross-site consistency checks.

### Data availability and access

Data generated from this study will be managed in accordance with DOD data governance policies. Deidentified datasets may be made available to external investigators through a controlled-access process, subject to DoD regulations and data use agreements.

### Sample size estimation

Power calculations for observational studies of this nature are typically based on the accuracy of the expected results [31,32]. These calculations were based on NHANES 2001–2018 population data that were weighted to match the military age range of 17 to 54 y. With an approximate *n* = 600, it is estimated that the number of SMs not meeting the estimated average daily requirement (EAR) for calcium [1000 mg/d, the same as the Military Dietary Reference Intakes (MDRI)] would be within 3% of the actual percentage of SMs not meeting the EAR for males and 2% for females. For vitamin D intake, with an *n* = 600, the estimate will be within 2% of the actual percentage of SMs not meeting the EAR for males and 1% for females. This study will detect within 2% of the actual percentage of male SMs and female SMs with high systolic blood pressure; within 2% of the actual percentage of male SMs and female SMs with high total cholesterol; within 2% of the actual percentage of male SMs and female SMs with high glucose; and within 1% of the actual percentage of male SMs and female SMs with elevated hemoglobin A1c.

### Statistical analyses

Statistical procedures will include the following: *1*) means, SEs, and distributions will be calculated for the entire sample for each variable; *2*) differences in means by sex, age, race/ethnicity, and rank will be computed and assessed with analysis of variance for statistical significance; *3*) percentages of SMs above, within, and below recognized cutoff levels will be calculated for laboratory values and dietary intake of vitamins and minerals, as defined by the DRIs and MDRIs; *4*) univariable and multivariable linear and logistic regression modeling of the associations between independent variables such as laboratory variables of nutritional status and outcomes such as cardiometabolic risk factors. The data will be analyzed using R and SAS 9.4 and will apply survey weights. In addition, actigraphy data will be processed and analyzed using the accelerometry-specific GGIR package for R (version 4.3.3). As new statistical methods emerge (e.g., machine learning), these methods may be applied to the dataset.

### Compensation

Participants received $400 for completing data collection during off-duty hours, because they cannot be compensated for research conducted during duty hours. This amount is prorated: $250 for completing the in-person visit, $50 for bringing medications and dietary supplements to the visit for tracking, $50 for collecting a stool sample, and $50 for completing the second dietary recall 3 to 10 d after the in-person visit.

## Discussion

This cross-sectional study will address major gaps in the available information on the health and nutritional status of the United States Army by providing comprehensive and representative data on dietary intake, nutritional status, and cardiovascular and metabolic health of active-duty Soldiers. These data will facilitate early detection of nutrient deficiencies, cardiometabolic risk, and disease prevalence. Furthermore, associative relationships of behavioral factors, particularly dietary intake, physical activity, and sleep, with nutritional and health biomarkers will be investigated. In addition, prospective analyses relating dietary intake and other factors to the development of diseases will be conducted using the SPHERE database. Ultimately, MHANES will guide evidence-based screening, education, and intervention strategies to promote healthy behaviors and inform H2F efforts to optimize well-being in active-duty Army personnel. Although the current study is focused exclusively on United States Army Soldiers, a long-term goal is to expand this research to other service branches and include additional sampling cycles similar to NHANES.

## Author contributions

The authors’ responsibilities were as follows – CEB, ASB, JCR, SRH, HRL: contributed to conceptualization; CEB, ASB, JCR, DAH, ACL, AMN, MEB, CW, TMR, DEB, CD, SBB, FHB, MPS, BST, CMS, SRH, HRL: contributed to data collection; CEB, ASB: contributed to writing; CEB: responsible for the final content of the manuscript; and all authors: contributed to the final review and read and approved the final version.

## Data availability

Data described in the manuscript, codebook, and analytic code will be made available upon request, pending application and approval.

## Disclaimer

The opinions or assertions contained herein are the private views of the authors and are not to be construed as official or as reflecting the views of the Army or the Department of War. Any citations of commercial organizations and trade names in this report do not constitute an official Department of the Army endorsement or approval of the products or services of these organizations.

## Declaration of Generative AI and AI-assisted technologies in the writing process

The authors declare that no generative AI or AI-assisted technologies were used in the writing of this manuscript.

## Funding

The study was funded by the Department of Defense, U.S. Army Medical Research and Development Command (USAMRDC) Broad Agency Announcement for Extramural Medical Research Program.

## Conflict of interest

The authors report no conflicts of interest.
